# Epigenetic Modifiers Affect the Bioactive Compounds Secreted by an Endophyte of the Tropical Plant *Piper longum*

**DOI:** 10.3390/molecules26010029

**Published:** 2020-12-23

**Authors:** Fuad Ameen, Abobakr Almansob, Mona Al Tami, Nouf Al-Enazi, Ahmed Al-Sabri, Raha Orfali

**Affiliations:** 1 Department of Botany and Microbiology, College of Science, King Saud University, Riyadh 11451, Saudi Arabia; aalmansob@ksu.edu.sa (A.A.); aalsbri@ksu.edu.sa (A.A.-S.); 2 Department of Biology, College of Science, Qassim University, Qassim 51452, Saudi Arabia; tamie@qu.edu.sa; 3 Department of Biology, College of Science and Humanities in Al-Kharj, Prince Sattam Bin Abdulaziz University, Al-kharj 11942, Saudi Arabia; n.alenazi@psau.edu.sa; 4 Department of Pharmacognosy, College of Pharmacy, King Saud University, P.O. Box 22452, Riyadh 11495, Saudi Arabia; rorfali@ksu.edu.sa

**Keywords:** endophytic fungi, *Phomopsis*, bioactivity, antagonistic, epigenetic modifier, small mass chemical

## Abstract

Seven endophytic fungi were isolated from the tropical medicinal plant *Piper longum* L. After preliminary screening, *Phomopsis heveicola* was selected for the epigenetic activation treatments. The antibacterial, antifungal, and antioxidant potentials of crude extracts obtained from the treatments (with and without epigenetic modifiers) were analyzed in vitro. The extracts inhibited growth of the human pathogens *Pseudomonas aeruginosa, Shigella sonnei, Streptococcus pyogenes*, and *Salmonella typhi*, as well as the phytopathogens *Puccinia recondita, Rhizoctonia solani, Phytophthora infestans*, and *Botrytis cinerea*. Furthermore, DPPH-scavenging activity was higher in valproic acid treated extracts. Volatile chemicals with known biological activities (measured with GC-MS/MS), were released in the valproic acid treatment. The antimicrobial potentials of the extracts were confirmed using MRM/MS analysis. The experiments revealed a new promising endophytic fungus, *P. heveicola*, to be utilized in biological plant protection and in biomedical applications.

## 1. Introduction

The development of biomedicine and biocontrol agents offers valuable alternatives to the excessive use of chemical drugs and control agents. The search for biological agents has involved several organisms, including plants that have been utilized in traditional biomedicine [1]. Recently, not only plants but their endophytic microorganisms have been found to produce a great variety of biologically active metabolites [2]. Such metabolites might be used, for instance, as antibiotics, anticancer agents, or even against plant pathogens and insects [3,4]. Some of the metabolites are volatile organic compounds (VOCs) that exist as mixtures of simple hydrocarbons, heterocycles, aldehydes, ketones, alcohols, phenols, thioalcohols, thioesters and their derivatives, benzene derivatives, and cyclohexanes [3]. Several VOCs produced by endophytic fungi have been shown to promote plant growth and vigor, and control plant pathogens [4].

Endophytic microorganisms that cooperate with their host plants produce metabolites that are transferred between the plant and the endophytes. Many endophytic species have been identified from different plant species and from different organs. *Fusarium tricinctum* has been isolated from the roots of *Panax notoginseng*; *Aspergillus oryzae* from *Raphanus sativus*; *Pseudomonas stutzeri* from the rhizosphere and endosphere of *Oryza sativa*; and *Penicillium canescens* from the roots, stems, and leaves of *Cajanus cajan*, to mention some examples [2]. The metabolite cocktails are unique and depend on several factors that are still largely unknown [5]. Even less is known about the mechanisms behind metabolite production [6]. It is known that many metabolites are silent (i.e., their genes are not expressed under laboratory conditions) [7]) but the development of epigenetics has allowed for great discoveries in this field [8]. Metabolites have been observed to be secreted when the organisms were treated with specific low-molecular-mass epigenetic modifiers [9,10]. The recently emerged research field of epigenetics has also shown the importance of epigenetic activation in microorganisms secreting metabolites [11,12,13]. The research has offered possibilities for novel biomedical applications. For example, the production of compounds against malaria and methicillin-resistant *Staphylococcus aureus* was enhanced when epigenetic modifiers were employed to activate the genes of the marine fungus *Leucostoma persooniimarine*, an endophyte of mangroves [14]. Similarly, epigenetic activation enhanced production of anti-inflammatory, antidiabetic, and antimicrobial compounds in several fungi [15,16,17,18,19]. However, their production for commercial use needs still to be developed. 

It has been suggested that every known fungus has the potential to produce antimicrobial compounds when treated with the right epigenetic modifiers [7]. However, further studies are needed to discover more species and novel antimicrobial substances, as concluded in a 2019 review by [12]. In their review, they showed that *Aspergillus* and *Fusarium* were the model genera in epigenetic studies for secondary metabolite production. The organisms were selected from similar habitats (mostly marine ecosystems). Consequently, in the present study we introduced a new habitat—a humid, tropical agricultural site—and a new plant species, *Piper longum* L.

The goal of the present study was to develop antimicrobial agents by taking advantage of the epigenetic activation of endophytic fungi isolated from *P. longum*, which is widely used in traditional Indian medicine to treat diseases such as tuberculosis, respiratory tract infections, dementia, epilepsy, and asthma [20]. *Piper longum* is known to release volatile metabolites such as piperine and piperidine [21]. However, production needs to be made more efficient for commercial use. Therefore, we isolated and identified endophytic fungi from *P. longum* and carried out experiments with two common epigenetic modifiers. We analyzed the crude extracts of *P. longum* grown in different culturing media for the fungal metabolites produced, as well as for its antimicrobial, antifungal, and antioxidant activities.

## 2. Results and Discussion

The endophytic fungus selected after antimicrobial screening showed 100% homology with *P. heveicola*, based on internal transcribed spacer (ITS) rRNA gene sequencing. The sequence was submitted to GenBank, NCBI, with the accession number MN857741.

### Bioactivity Analysis

The crude extracts of the controls without epigenetic modifiers had weak antibacterial activity against *P. aeruginosa, S. sonnei*, and *S. pyogenes*, and weak antifungal activity against *R. solani* and *P. infestans*. The rest of the pathogens were not inhibited by the crude extracts.

In the two-way ANOVA of each pathogenic species, there were significant interactions between the epigenetic modifiers and their concentrations, indicating that the effects of the modifiers depended on their concentrations. The antimicrobial activities of the crude extracts of *P. heveicola* varied significantly depending on the epigenetic modifier and the concentration at which it was used. For a detailed inspection, *t*-tests were carried out to compare each concentration of epigenetic modifier separately to the respective positive control within the pathogenic species. In some cases, the positive control had more antimicrobial activity than the tested *P. heveicola* extract. Only antimicrobial effects higher than the positive controls were tested and reported.

In general, the valproic acid treatment resulted in higher antibacterial and antifungal activities than the 5-azacytidine treatment. In the *t*-tests, 5-azacytidine had no significant effect on either antimicrobial or antifungal activity compared to the positive controls.

**Figure 1:**
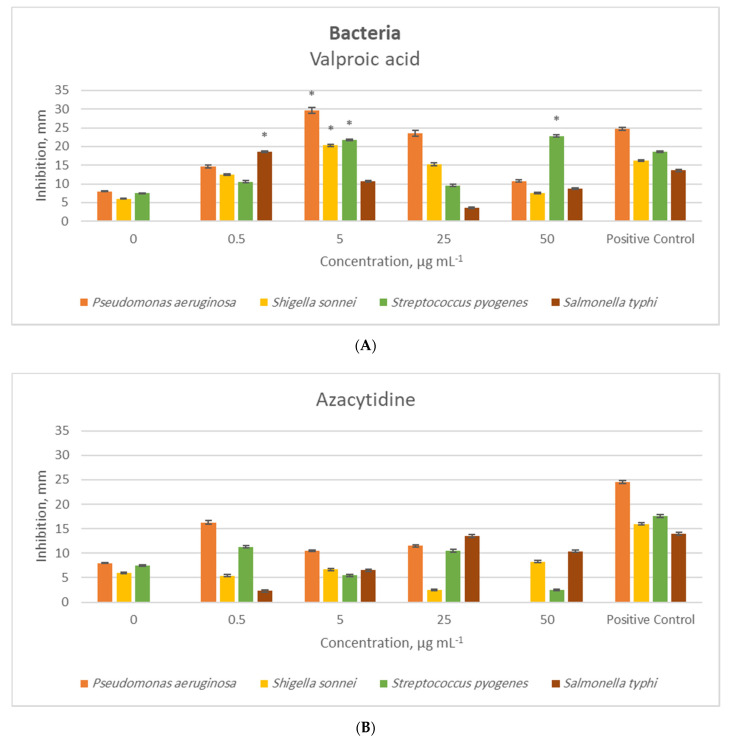
Antibacterial activity (zone of inhibition, mean, and SD error bars, *n* = 3) of the crude extract of the endophytic fungus *P. heveicola* cultured with different concentrations of the epigenetic modifiers valproic acid (**A**) and 5-azacytidine (**B**), where 0 µg/mL refers to the control treatment. The positive control contains ampicillin + tetracycline. * indicates significant difference (*t*-test, *p* < 0.05) from the positive control.

Shown using a *t*-test, valproic acid had a significantly higher antibacterial efficiency than the positive controls in some cases (Figure 1). The lowest concentration valproic acid treatment (0.5 μg/mL) presented a 37% higher inhibition zone against *S. typhi* than the positive control. Valproic acid at a concentration of 5 μg/mL had 20%, 25%, and 17% higher inhibition zones against the bacteria *P. aeruginosa*, *S. sonnei*, and *S. pyogenes*, respectively, compared to the positive controls. The highest concentration of valproic acid tested (50 μg/mL) had a 23% higher inhibition zone against *S. pyogenes* than the positive control.

**Figure 2:**
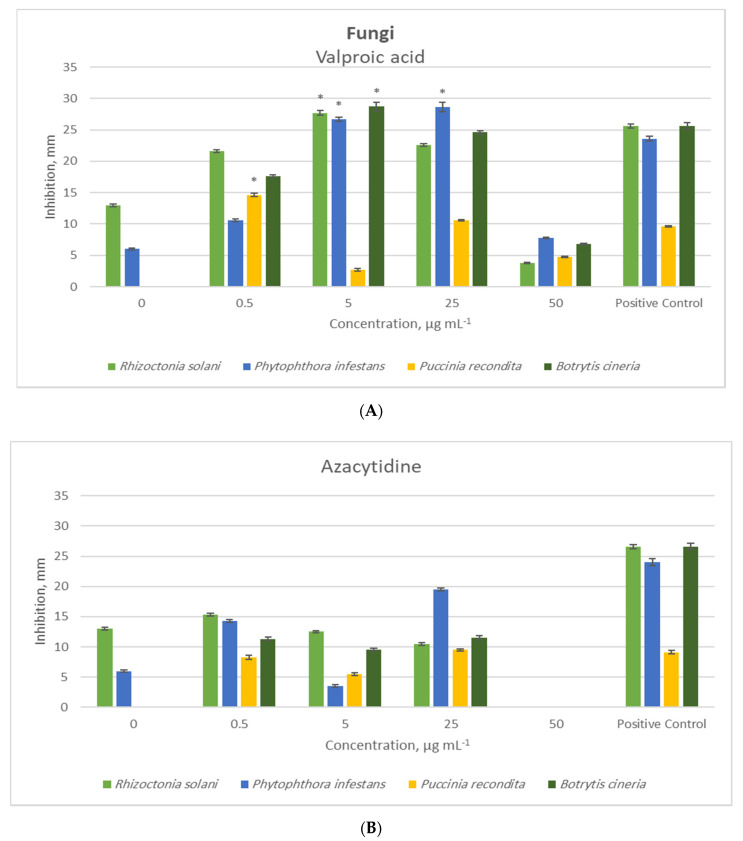
Antifungal activity (zone of inhibition, mean, and SD error bars, *n* = 3) of the crude extract of the endophytic fungus *P. heveicola* cultured with different concentrations of the epigenetic modifiers valproic acid (**A**) and 5-azacytidine (**B**), where 0 µg/mL refers to the control treatment. The positive control contains gentamycin + fluconazole. * indicates significant difference (*t*-test, *p* < 0.05) from the positive control.

In the *t*-tests of antifungal activity, 5-azacytidine had no increasing effects on the inhibition zones when compared to the positive controls (Figure 2). The lowest concentration valproic acid treatment (0.5 μg/mL) caused a 21% higher inhibition zone against *P. recondite* than the positive control. Valproic acid at 5 μg/mL resulted in 8%, 13%, and 12% higher inhibition zones against *R. solani*, *P. infestans*, and *B. cineria*, respectively, compared to the positive controls. Valproic acid at 25 μg/mL resulted in a 21% higher inhibition zone against *P. infestans* than the positive controls. The highest concentration of valproic acid did not have a significant increasing effect on antifungal activity.

The antioxidant potential is a useful measure to assess the biologically active metabolites. Antioxidants scavenge free radicals minimizing their adverse effects. In this study, we assessed the antioxidant potential with a common technique DPPH-scavenging activity [22]., which increased along with the epigenetic modifier concentration used (Table 1) similar to positive control rosmarinic acid. The valproic acid treatment increased DPPH activity more than the 5-azacytidine treatment. At their lowest concentrations, valproic acid had a low DPPH radical–scavenging activity (3%), while 5-azacytidine had a comparatively high (21%) scavenging activity. However, at the highest concentrations, the respective values were 71% and 60%.

**Figure 3:**
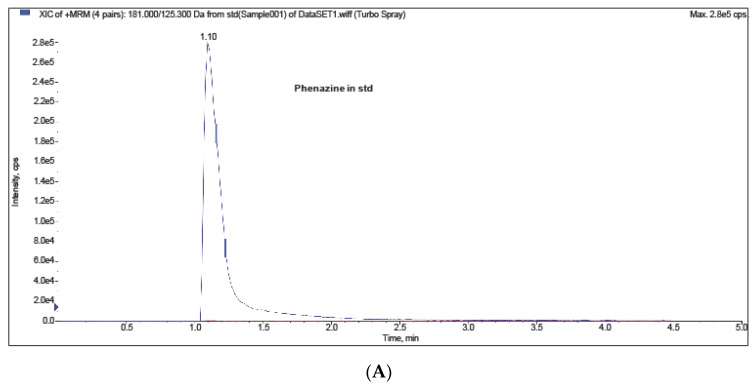

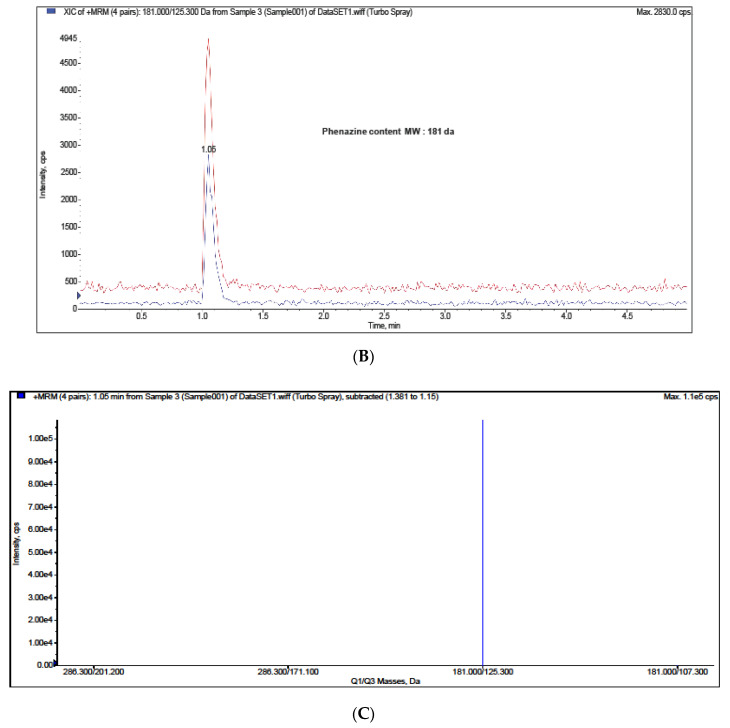
MRM-MS of phenazine-1-carboxylic acid (C_12_H_8_N). (**A**) Standard peak was detected in the retention time of 1.10 min at a wavelength of 248 nm. (**B**) The endophytic fungal valproic acid treated extract also peaked at the retention time of 1.05 min. (**C**) MRM-MS detected that the extract of endophytic fungus contained a mass at 181 kDa (positive mode of ionization). The mass of phenazine-1-carboxylic acid is 180 kDa.

The presence of the antibiotic compound phenazine-1-carboxylic acid was observed with a multiple reaction monitoring mass spectrometry (MRM-MS) system. The standard peak was detected in the retention time of 1.10 min (Figure 3A), which was similar to the valproic acid treatment (1.05 min) (Figure 3B). No other treatments were detected at the peak. The extract from the valproic acid treatment resulted in a mass of 181 kDa (positive mode of ionization), while the mass of phenazine-1-carboxylic acid was 180 kDa (Figure 3C).

MRM-MS analysis confirmed that the endophytic fungus *P. heveicola* produced antibiotics after valproic acid treatment. Phenazine and its derivatives are heterocyclic nitrogen compounds that have been reported to have activity against *Gaeumannomyces* sp., *Fusarium* sp., *Pythium* sp., *Rhizoctonia solani, Gibberella* spp., *Drechslera graminea*, and *Alternaria* spp. [23]. These compounds have mainly been detected from a *Pseudomonas* sp. [24] and a *Streptomyces* sp. [25], whereas endophytic fungi–derived phenazine derivatives are rarely reported. An endophytic fungus, *Nigrospora oryzae*, isolated from *Coccinia grandis* has been reported to produce phenazine-l-carboxamide [26]. Our study can add the endophytic *P. heveicola* isolated from *P. longum*.

**Figure 4:**
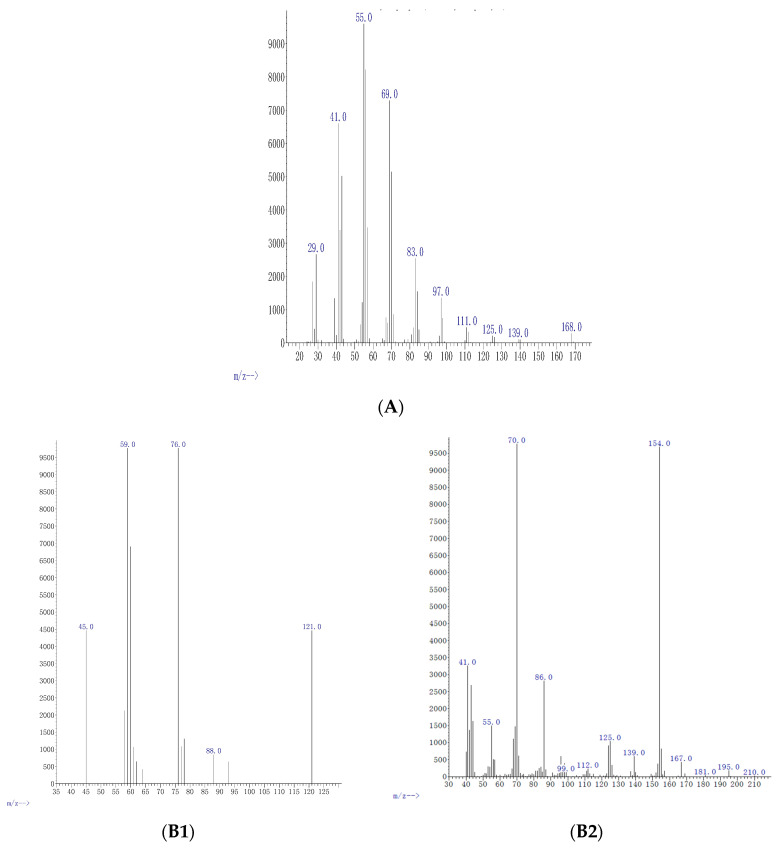

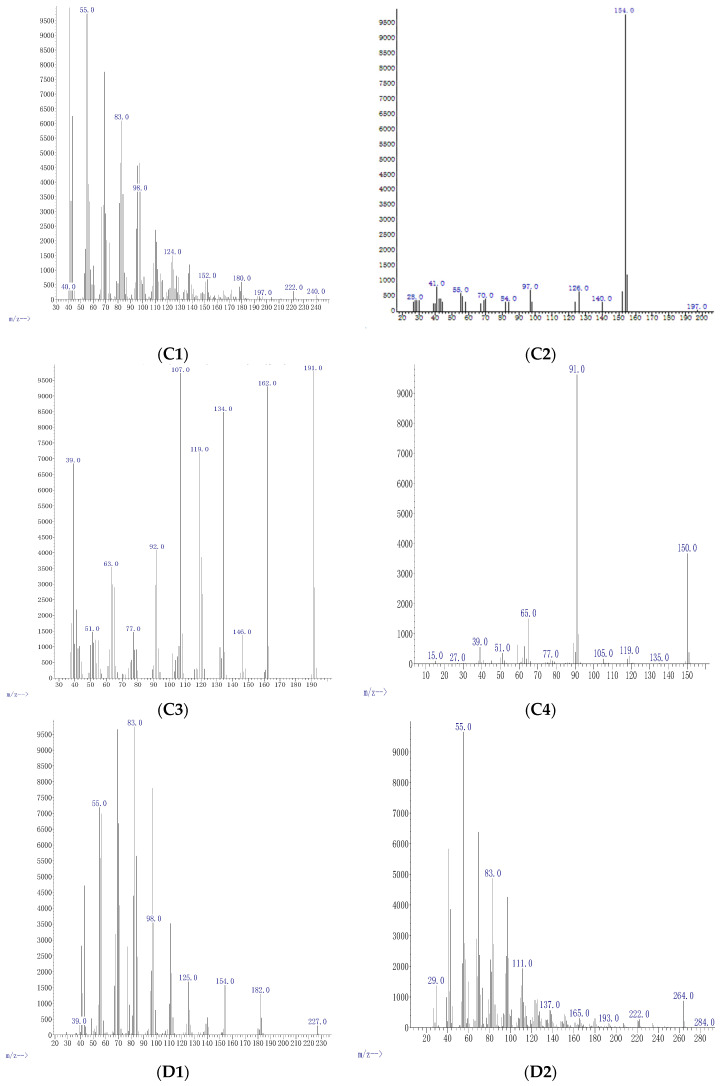
GC-MS/MS mass spectra of the crude extract prepared from the medium of the endophytic fungus *P. heveicola* cultured with valproic acid as an epigenetic modifier at different concentrations. (**A**) 0.5 μg/mL cyclopropane. (**B**) 5 μg/mL: Oxathiazole 2 thione (**B1**) and pyrrolo[-1,2-a] pyrazine-1,4-dione,hexahydro-3,2-methyl (**B2**). (**C**): 25 μg/mL cyclopentadecanone (**C1**); oxazole (**C2**), 4-ethyl 4,5-dihydro 2,2-hydroxy phenyl (**C3**); and benzene acetic acid (**C4**). (**D**) 50 μg/mL chloroacetic acid (**D1**) and cis-vaccenic acid (**D2**).

The crude extract of the control treatment and the extracts of the 5-azacytidine treatments contained no valuable chemicals (data not shown). However, the valproic acid treatments resulted in several chemicals in the crude extracts, depending on the valproic acid concentration used. The lowest concentration of valproic acid tested (0.5 μg/mL) contained cyclopropane (C_3_H_6_) with 4.9% relative abundance (Figure 4A). The concentration of 5 μg/mL led to oxathiazole 2 thione (C_2_H_3_NOS_2_) and pyrrolo [1,2-a]pyrazine-1,4-dione,hexahydro-3,2-methyl (C_11_H_18_N_2_O_2_) with relative abundances of 3.8% and 24%, respectively (Figure 4B). Cyclopentadecanolide (C_15_H_28_O_2_); N-allyldecylamine (C_13_H_27_N); oxazole, 4-ethyl-4,5-dihydro-2-(2-hydroxyphenyl)-(C_11_H_13_NO_2_); and benzyl ethanoate (C_9_H_10_O_2_) were present in the extracts of 25 μg/mL of valproic acid treatment with relative abundances of 14%, 38%, 55%, and 48%, respectively (Figure 4C). Diphenan (C_14_H_13_NO_2_) and methyl 2,3-anhydro-4,6-*O*-benzylidenehexopyranoside (C_14_H_16_O_5_) were detected in the crude extract of 50 μg/mL concentration with 24% and 37% relative abundances, respectively (Figure 4D) (Table 2).

All the biological activities studied depended on the epigenetic modifier and concentration used. We interpreted that 5-azacytidine did not act as an effective epigenetic modifier, in contrast to valproic acid that was an effective epigenetic modifier of endophytic *P. heveicola*. We showed that the histone deacetylase inhibitor (valproic acid) had the ability to activate silent pathways of the endophytic fungus *P. heveicola*, whose metabolites had remarkable activity against both human and plant pathogens. The metabolites inhibited the growth of four clinical human pathogenic bacteria, namely *P. aeruginosa, S. sonnei, S. pyogenes*, and *S. typhi*. The metabolites also inhibited four plant pathogenic fungi, namely *R. solani, P. recondita, P. infestans*, and *B. cineria.* The antioxidant assays gave somewhat different results because both epigenetic modifiers seemed to induce antioxidant activity of *P. heveicola* medium extracts. However, antioxidant potential increased remarkably along with the epigenetic modifier concentration only in valproic acid treatments. Therefore, we concluded that valproic acid was a more effective epigenetic modifier than 5-azacytidine.

Our experiment with different concentrations of epigenetic modifiers revealed great variation in biological activities depending on the concentration of the epigenetic modifier used. In some cases, the positive controls had more antimicrobial activity than the treated *P. heveicola* extracts, indicating that the antibiotics and fungicides were more effective than the biological agents. These great differences between the pathogenic species and the effective concentrations of valproic acid call for further detailed studies. However, in all cases, valproic acid showed its ability to act as an effective epigenetic modifier for endophytic *P. heveicola* and should be studied further.

Both epigenetic modifiers we used have previously been observed to induce metabolite secretion in certain organisms to help the overexpression of specific genes [18]. Endophytic fungi *Leucostoma persoonii*, *Alternaria* sp., and *Pestalotiopsis acacia* have been reported to be induced by 5-azacytidine [14]. Valproic acid was reported as an efficient epigenetic modifier of endophytic *Nigrospora sphaerica* [35]. Valproic acid was also observed to induce the endophytic fungus *Aspergillus fumigatus* [36]. In that study, 5 μg/mL of valproic acid was the most effective concentration, resulting in a tenfold increase in the production of bioactive compounds. In our study, the same concentration also appeared to be the most efficient. However, we observed differences between the pathogens and, therefore, we suggested that different concentrations induced production of different chemicals. One possibility to improve metabolite secretion might be to combine different epigenetic modifiers. For instance, [37] found that using valproic acid in combination with procaine induced metabolite production of a *Xylariaceae* species.

Biological properties of chemical compounds—such as Oxathiazole 2 thione; pyrrolo[1,2-a]pyrazine-1,4-dione, hexahydro-3-(2-methyl); cyclopentadecanolide, *N*-allyldecylamine 1-decanamine; oxazole, 4-ethyl-4,5-dihydro-2-(2-hydroxyphenyl)-; benzyl ethanoate; diphenan; and methyl 2,3-anhydro-4,6-*O*-benzylidenehexopyranoside)—have previously been reported to have high free-radical-scavenging, antifungal, and antibacterial activity in the PubChem database (PubChem is a registered trademark of the U.S National Library of Medicine). For the first time, the compounds were observed to be released by *P*. *heveicola* isolated as an endophyte of *P. longum*.

*P. longum* has been studied for its antimicrobial metabolites before. The endophytic fungus *Periconia* sp. was reported to produce piperine (5-(3,4-methylenedioxyphenyl)-1-piperidinopent-2,4-dien-1-one) that displayed antibacterial activity against *Mycobacterium tuberculosis* and *M. smegmatis* [38]. More often than *P. longum*, another *Piper* species called *P. nigrum* and its endophytes have been studied for their piperine release, as reviewed recently by [39]. Reference [40] reported that the endophytic fungus *Colletotrichum gloeosporioides* from *P. nigrum* also released plant specific metabolite piperine.

We isolated and identified *P. heveicola* as an endophyte of *P. longum*. Many different *Phompsis* species have been reported as phytopathogens and sources of potential biological control agents [40]. *P. cassie* was reported as an endophytic fungus that released bioactive compounds in the plant *Cassia spectabilis* [41]. The endophytic fungus of rice *P. liquidambari* had a role in nitrogen transformations by improving the nutrient uptake of rice [41]. Previous reports on *P. heveicola* are scarce. *P. heveicola* was reported as an endophyte of mangroves in India [42]. The species was also reported as a coffee pathogen in China [43]. However, we did not find reports regarding the biologically active metabolites of *P. heveicola*.

## 3. Materials and Methods

### 3.1. Endophytic Fungi Isolation from Piper longum L.

Twenty *Piper longum* plants were collected from the Rama Farm, Al-Qassim, Saudi Arabia (26°5′38.7168″ N and 43°58′24.4344″ E) and stored at 4 °C. For surface sterilization, the plant stems and roots were soaked successively in 70% ethanol (1 min), 3% sodium hypochlorite (3 min), and 70% ethanol (30 s) as described by [44]. Finally, the ethanol was removed using sterile distilled water. The disinfection efficiency was checked by adding the final rinse onto Petri plates containing potato dextrose agar (PDA) (200 g potato extract, 20 g dextrose, 15 g agar, pH 6.5) and Manihot dextrose agar (MDA) (200 g cassava, 20 g dextrose, 15 g agar, pH 6.5). The extracts of potato and cassava were prepared by using 200 g of the plants cut into small pieces and boiled. Dextrose was added to the infusion and the pH was adjusted using 1 M HCl.

The stems of the plants were cut into 4–6 mm pieces and placed onto Petri dishes containing PDA or MDA supplemented with both tetracycline (50 μg/mL) and streptomycin (50 μg/mL) to inhibit bacterial growth. The plates were incubated at 28 °C, and when the fungal mycelium appeared after 5–6 days, the fungi were transferred onto new PDA and MDA plates and incubated again for seven days. After incubation, the plates were stored at 4 °C for further study. Primary identification of the isolates was carried out using the Lactophenol Cotton Blue Staining method [45] and scanning electron microscopy as described by [46]. A primary screening of seven morphologically different fungi was carried out using an antibacterial assay (explained below). The species with antibacterial activity were chosen for further investigation.

### 3.2. Molecular Identification of the Endophytic Fungi

Potato dextrose broth (PDB) (2 μL) was added into endophytic fungal culture tubes and vortexed to collect spores (1 × 10^9^ spores/mL). Then, the PDB and spores were poured into 100 mL flasks containing only PDB and incubated on a rotary shaker at 150 rpm for three days at room temperature. After that, the mycelium was filtered and frozen at −80 °C for 30 min and lyophilized. The mycelium was ground with liquid nitrogen using a sterile mortar to obtain mycelium powder. DNA was extracted using a DNeasy fungal mini kit. The ITS regions of the DNA (ITS1: 5′-TCCGTAGGTGAACCTGCGG-3′ and ITS4: 5′-TCCTCCGCTTATTGATATGC-3′) were amplified by PCR using the following thermal cycles: initial denaturation at 95 °C for 5 min, denaturation with 35 cycles at 94 °C for 1 min, annealing at 55 °C for 30 s, extension at 72 °C for 2 min, and a final extension at 72 °C for 10 min. Sequencing was carried out using a BigDye Terminator sequencing kit (Applied Biosystems, Foster City, CA, USA). The alignment and phylogeny reconstructions were performed with the ‘build’ function of ETE3 v3.1.1(EMBL, Heidelberg, Germany) [47] as implemented on the Genome Net (https://www.genome.jp/tools/ete/).

### 3.3. Epigenetic Modifier Experiments

Two commonly used and efficient epigenetic modifiers, valproic acid and 5-azacytidine, were tested at four concentrations (0.5, 5.0, 25, and 50 μg/mL) based on previous studies [18]. First, the media were prepared in 500 mL Erlenmeyer flasks containing 200 mL PDB and either valproic acid or 5-azacytidine as three replicates. Control PDB cultures were prepared without epigenetic modifiers. The broths were inoculated with freshly grown cultures of the endophytic fungus and incubated at 28 °C ± 2 °C on a rotary shaker at 110 rpm for 30 days.

After incubation, the broths were filtered through Whatman No. 1 filter paper, and the collected supernatants were used for crude metabolite extraction. First, the supernatants (200 mL) were added into a separating funnel with an equal amount of ethyl acetate and shaken vigorously for 20 min. The organic phase was collected into a 500 mL conical flask. The first step was repeated twice more. The crude extract in the organic phase was first evaporated in a rotatory evaporator (IKA, Königswinter, Germany) and finally dried in a fume hood. The crude compounds were weighed, dissolved in methanol, filtered through a 13 mm syringe filter with pore size 2 µm 13 mm pore size syringe filter, and stored at 4 °C.

### 3.4. Bioactivities

The crude extracts from the epigenetic modifier experiments and controls were used to perform antimicrobial activity assays. For this, sterile paper discs were impregnated with the crude extracts (20 mg/mL). The antibacterial test was carried out using a modified Kirby–Bauer method [48], where paper discs impregnated with ampicillin (5 μg/mL) and tetracycline (5 μg/mL) were used as positive controls. Four clinical human bacterial pathogens, namely *P. aeruginosa*, *S. sonnei*, *S. pyogenes*, and *S. typhi* (provided by the Department of Botany & Microbiology, College of Science, King Saud University, Riyadh, Saudi Arabia), were inoculated into Mueller–Hinton agar plates. The paper discs with the crude extracts and positive controls were placed onto individual plates, which were then incubated overnight at 37 °C. After that time, the inhibition zones were measured.

The antifungal activity assay used was modified from the method of [49]. First, four plant pathogenic fungi—namely *B. cineria*, *R. solani*, *P. recondite*, and *P. infestans* (preserved at King Saud University)—were inoculated on PDA agar plates. Then, the paper discs impregnated with the crude extracts, PDA controls, and positive controls (5 μg/mL gentamycin and 5 μg/mL fluconazole) were placed onto PDA plates, 4 cm away from the pathogenic cultures. The plates were incubated at 25 °C for five days before the inhibition zones were measured.

The antioxidant potentials of the crude extracts were assessed using DPPH assays, which measured the free-radical-scavenging activity, as described by [50]. The samples (2 mL) such as the crude extract, positive control rosmarinic acid (Sigma-Aldrich Co, Saint Louis, MI, USA), and negative control glucose (Sigma-Aldrich Co, Saint Louis, MI, USA) were mixed with DPPH (4 mL) and incubated at 37 °C in the dark for 30 min before the absorbance was measured at 517 nm using a spectrophotometer. Scavenging activity was calculated as the percentage of sample absorbance from that of the control (DPPH added in the crude extract of the medium with no epigenetic modifier). A lower percentage indicated higher scavenging activity.

### 3.5. Chemical Analyses

The crude extracts were analyzed with GC-MS/MS (fused silica 15 m × 0.2 mm ID × 1 μm of capillary column, Shimadzu GC-MS QP, Kyoto, Japan) and the sample injection volume was 1 μL. The settings were: initial temperature 30 °C for 2 min, gradual rise to 280 °C and maintenance for 9 min, ionization voltage 70 eV, and mass spectral scan range 25–250 m/z. The GC-MS/MS data were interpreted with the NIST database performed with MMF (minimum match factor, NIST, Gaithersburg, MD, USA) 75 for all the components and also using the virtue of comparisons having more than 62,000 patterns.

MRM/MS analysis was used to detect the antibiotic compound phenazine-1-carboxylic acid (C_12_H_8_N) in the extracts. Agilent 6420 Triple Quad LC/MS system with C18-AR column, 3 µm, 50 × 4.6 mm (MAC-MOD Analytical Inc. Part No. EXL1190546U, MAC-MOD Analytical, Chadds Ford, PA, USA) was used with the mobile phase as acetonitrile-water-trifluoroacetic acid (90:10:0.04, *v*/*v*/*v*) at flow rate 1.0 mL/min and wavelength 248 nm and the sample injection volume was 1 μL.

### 3.6. Statistical Analyses

Two-way ANOVAs were carried out with epigenetic modifiers and concentrations as factors. If significant interactions were found, *t*-tests were carried out to analyze the effects of each concentration of epigenetic modifier in comparison to their respective positive controls. The tests were carried out separately for each of the eight pathogenic species. The data were log-transformed when necessary. Differences with *p* < 0.05 were considered significant.

## 4. Conclusions

We can recommend that the endophytic fungus, *P. heveicola* can be utilized in biological plant protection and biomedicine. The addition of the epigenetic modifier, valproic acid, will greatly enhance the release of volatile bioactive metabolites. In contrast, 5-azacytidine is not an effective epigenetic modifier. In the tests, the antimicrobial efficiency depended largely on the concentration of valproic acid used, as it was shown that different compounds were released in different concentrations. Therefore, more complex study designs should be carried out to determine the optimal conditions for efficient production of these biomedical agents. For instance, mixing different epigenetic modifiers with different concentrations would be of interest. We can recommend that the endophyte of *P. longum* can be used to produce biologically active metabolites for biocontrol purposes.

## Figures and Tables

**Table 1:** Antioxidant potential assessed as DPPH radical scavenging activity (percentage of control with no epigenetic modifier addition, mean ±SD, *n* = 3) of the crude extract of *P. heveicola* cultured in medium with different concentrations of two epigenetic modifiers. In addition, included positive control roamarinic acid and negative control glucose.

	Epigenetic Modifier			
Concentration, μg mL^−1^	Positive Control	Negative Control	Valproic Acid	Azacytidine
0.5	23 ± 1	1 ± 2	3 ± 2	21 ± 5
5	42 ± 1	1.4 ± 2	28 ± 9	51 ± 8
25	73 ± 3	1 ± 2	68 ± 5	58 ± 7
50	83 ± 1	1.3 ± 2	71 ± 8	60 ± 6

**Table 2:** Relative abundance, molecular mass, and fragments of the compounds present in the endophytic fungus *P. heveicola* extract cultured with the epigenetic modifier valproic acid in different concentrations (μg mL^−1^) as identified using GCMS/MS analysis.

Conc μg mL^−1^	Compound	Formula	M Da	Fragments	Relative Abundance %	% of Agreement with NIST Database	Functional Uses
0.5	Cyclo-propane	C_3_H_6_	42.08	29, 41, 42, 55, 69, 83, 97, 111, 125, 139, 168	4.9	98%	Anesthetic [27]
5	Oxathiazole 2 thione	C_2_H_3_NOS_2_	121.19	45, 59, 78, 88, 121	37	97%	Anti-bacterial agent [28]
5	Pyrrolo[1,2-a] pyrazine-1,4-dione, hexahydro-3-(2-methyl)	C_11_H_18_N_2_O_2_	210.27	41, 55, 70, 86, 99, 112, 125, 139, 154, 167, 181, 196, 210	3.7	99%	Anti-cancerous/anti-oxidant [22]
25	Cyclopentadecanolide	C_15_H_28_O_2_	240.38	40, 55, 83, 98, 124, 152, 180, 197, 222, 240	33	98%	Flavouring agent [29]
25	N-allyldecylamine 1-Decanamine -	C_13_H_27_N	197.36	28, 41, 55, 70, 84, 97, 126, 140, 154, 197	5.0	97%	Anti atherosclerosis [30]
25	Oxazole, 4 ethyl 4,5 di hydro 2, 2hydroxy phenyl	C_11_H_13_NO_2_	191.23	39, 51, 63, 77, 92, 107, 119, 134, 146, 162, 191	85	97%	Anti-bacterial [31]
25	Benzyl ethanoate	C_9_H_10_O_2_	150.17	15, 27, 39, 51, 77, 91, 105, 119, 135, 150	3.8	99%	Flavouring agent [32]
50	Diphenan	C_14_H_13_NO_2_	227.26	39, 55, 83, 98, 125, 154, 182, 227	14	98%	Administrated for the treatment of pinworms [33]
50	Methyl 2,3-anhydro-4,6-*O*-benzylidenehexopyranoside	C_14_H_16_O_5_	264.27	29, 55, 83, 111, 137, 165, 193, 222, 264, 284	1.3	98%	Antioxidant [34]
